# A Comparison of Hemostatic Activities of Zeolite-Based Formulary Finishes on Cotton Dressings

**DOI:** 10.3390/jfb14050255

**Published:** 2023-05-04

**Authors:** J. Vincent Edwards, Nicolette T. Prevost, Michael Santiago Cintron

**Affiliations:** Southern Regional Research Center, United States Department of Agriculture (USDA), Agricultural Research Service, New Orleans, LA 70124, USA

**Keywords:** cotton, zeolites, hemostatic wound dressings, fabric formulations

## Abstract

The need for affordable effective prehospital hemostatic dressings to control hemorrhage has led to an increased interest in new dressing design approaches. Here we consider the separate components of fabric, fiber, and procoagulant nonexothermic zeolite-based formulations on design approaches to accelerated hemostasis. The design of the fabric formulations was based on incorporation of zeolite Y as the principal procoagulant, with calcium and pectin to adhere and enhance the activity. Unbleached nonwoven cotton when combined with bleached cotton displays enhanced properties related to hemostasis. Here, we compare sodium zeolite with ammonium zeolite formulated on fabrics utilizing pectin with pad versus spray-dry-cure and varied fiber compositions. Notably, ammonium as a counterion resulted in shorter times to fibrin and clot formation comparable to the procoagulant standard. The time to fibrin formation as measured by thromboelastography was found to be within a range consistent with modulating severe hemorrhage control. The results indicate a correlation between fabric add-on and accelerated clotting as measured by both time to fibrin and clot formation. A comparison between the time to fibrin formation in calcium/pectin formulations and pectin alone revealed an enhanced clotting effect with calcium decreasing by one minute the time to fibrin formation. Infra-red spectra were employed to characterize and quantify the zeolite formulations on the dressings.

## 1. Introduction

Since the advent of hemorrhage control dressing research and development, design concepts have been directed to a variety of mechanistic targets to promote accelerated hemostasis based on the employment of pro-coagulants, mucoadhesives, or coagulation factor concentrators [1,2]. Moreover, hemostatic material types are usually characterized as accelerating surface hemostasis in these categories [1,2,3]. The focus of the mechanism of action is usually the intrinsic coagulation pathway and its key serine proteases (Factor XII, Fibrinogen, Factor VII) that mediate clotting through contact with externally charged surfaces or promotion of platelet adhesion [4,5]. Treated dressings are typically woven or nonwoven textile materials that have a hemostatic agent incorporated, i.e., clay minerals, chitosan (used singularly as a fiber or coating), modified polysaccharides, and fibrin sealant as the active clotting agent [3]. Notable as well are materials that promote hemostatic activity based on single or multiple fiber blends [6,7,8].

Advances in hemostatic control and lessons learned through technologies developed have been documented in recent years [9]. Ideally, a hemostatic dressing has the ability to rapidly bring a hemorrhaging vessel under control (within 2 min). It should be lightweight and durable, have a prolonged shelf-life even under extremes of temperature, not pose additional risks, and, finally, be inexpensive. The criteria for material selection should be based on probability of success in vivo, stability, ease of use, and ease of manufacturing. No current hemostatic agents meet all the requirements. There are continuous efforts to develop new hemostatic agents with various delivery mechanisms for treating severe hemorrhage in a military setting [10]. Thus, the development of hemorrhage control dressings has followed a path through the years of discovery and application of procoagulant materials to dressing motifs.

Among the types of procoagulants employed the use of aluminosilicates applied to different substrate fabrics has been explored in an effort to close the gap in meeting these criteria [11]. For example, the formation of tightly bound zeolite crystals on cotton fibers has been shown to be an effective hemostat and coating with hydrophobic paraffin improved adhesion issues [12,13]. Historically, zeolite was first identified as an advance in the use of aluminosilicates for hemorrhage control [14]. It has high absorbance and functions as a concentrator of platelets and clotting factors. Limitations in the safety of zeolite and related aluminosilicate minerals have been addressed for the exothermic reaction and thrombosis found to prohibit use of an early version [15]. Nonetheless, increased interest in the interaction of blood proteins as fibrinogen with zeolite are beginning to shed light on the molecular aspects of coagulopathic mechanisms [16]. Here we consider a non-exothermic zeolite formulation in combination with a cotton-based dressing.

Here, we have considered the initiation of hemostasis through the synergism of a cotton-based dressing possessing properties of high absorption capacity, a potent procoagulant zeolite, and the formulary application of pectin and calcium as adjuvants to both facilitate adherence and promote hemostasis. In addition, the paper explores the effect of different types of finishing chemistry approaches to understand the structure/function relations of the zeolite/cotton formulations on functional clotting as measured by thromboelastography and to employ FTIR to delineate the molecular connectivity of the formulations and within the zeolite/pectin/cotton fabric and fiber structure.

## 2. Materials and Methods

### 2.1. Materials

CBV100 and CBV300 zeolites, purchased from Zeolyst, are the synthetic faujasite Y zeolite with the sodium cation (NaY) and the ammonium cation (NH_4_Y), respectively. The SiO_2_/Al_2_O_3_ molar ratios are 4.9–5.4 and 5.1 for NaY and NH_4_Y, respectively. All other chemicals and fabrics were from existing supply/inventory. The pectin (PEC), from citrus peel (≥74% galacturonic acid), l-ascorbic acid, and calcium chloride (CaCl_2_), was purchased from Sigma Aldrich (now Millipore Sigma). Sodium hypophosphite monohydrate (NaH_2_PO_2_ ·H_2_O) was purchased from JT Baker. Ultrapure water (18 Ω), Millipore, was used as solvent. The fabrics used were as follows: TACGauze (TGz) from H&H Medical, a blend of 60% greige cotton/20% bleached cotton/20% polypropylene; Fine Mesh Gauze (FMGz), 100% bleached cotton (# 4-2915 36in wide 50 yds roll from DeRoyal); Hydroentangled nonwoven fabric (NW85), 100 bar 85% true cotton (greige cotton) and 15% bleached cotton (true cotton). For spray application of formulation, an Aldrich-flask type thin-layer chromatography (TLC) sprayer was used.

### 2.2. Fabric Treatment Methods

#### 2.2.1. Application Method 1: Pad-Dry

To treat the fabrics, fabric swatches were submersed and saturated in a solution volume of 20× the weight of fabric. The saturated swatches were padded with a hand-cranked wringer (Calliger). Padding was repeated, and the wet padded weight of the swatch was recorded. The swatches dried on a screen or metal frame in a force draft oven of either 100–105 °C for 5–10 min or 120 °C for 3–5 min without tension. Swatches were not rinsed. The formulation swatches equilibrated overnight before weight measurement. In a two-step pad-dry method, the fabric swatches were padded with a weight percent solution of CaCl_2_ and dried for 3 min at 100 °C and then padded with weight percent pectin and zeolite solution and dried at 105 °C or 120 °C for 5 min. Swatches equilibrated overnight at ambient conditions and weighed.

#### 2.2.2. Application Method 2: Pad-Spray

Fabric swatches were saturated with a weight percent CaCl_2_ solution volume 20 times its weight and were padded to remove excess solution. Then, swatches were clipped to a frame and sprayed with a percent solution of pectin and zeolite previously vortexed using an aspirator (attached to house air). Spraying application was in a sweeping motion and applied to one-side of fabric surface. The fabric formulation was dried for 5 min at 105 °C. Without rinsing, fabrics were equilibrated overnight to room atmosphere and humidity and then weighed.

### 2.3. Fourier-Transform Infrared Spectroscopy

FTIR examinations of TGz, NaY, and NH_4_Y film samples were performed using a Vertex 70 FT-IR spectrometer (Bruker Optics, Billerica, MA, USA) using the attenuated total reflection (ATR) sampling accessory (Pike Technologies, Madison, WI, USA). Samples were placed on top of diamond-ZnSe reflective ATR crystal and secured with a metal clamp. Each sample was examined at 4 different points chosen at random. A total of 32 scans were measured between 3800 and 600 cm^−^^1^ with a resolution of 4 cm^−^^1^ for each replicate. Spectra are presented as the average of four replicates and corrected for baseline and normalized as stated in each figure. No ATR correction or atmospheric compensation was performed. For the NaY and NH_4_Y film studies, samples were dried overnight in a 105 °C oven and allowed to reach room temperature in a desiccator before each examination. Spectral figures were prepared using OriginPro 2019b (OriginLab Corporation, Northampton, MA, USA).

### 2.4. Thromboelastography

Thromboelastography (TEG) assesses the viscoelastic properties of whole blood under low shear conditions and provides information about global hemostatic function from the beginning of clot formation through clot retraction and fibrinolysis [17]. Thromboelastography was performed at 37 °C on a TEG 5000 Thromboelastograph^®^ Hemostasis Analyzer System using the TEG analytical software 4.2.3 (Haemonetics Corporation, Niles, IL, USA). Twenty microliters of citrated saline (5.375 mM disodium citrate, 146 mM NaCl) were added to the disposable cup in each channel, 2 total, containing a fabric sample circle, cut 3/16 inches in diameter, using an Osborne Arch Punch. To this, 30 µL calcium chloride (0.2 M) and 310 µL citrated bovine blood were added, and the analysis was started immediately. Multiple runs were performed on each sample and subject to Excel statistical analysis using the Descriptive Analysis.

### 2.5. Scanning Electron Microscopy (SEM)

The imaging of the nonwoven fabrics’ morphology and fine structure was conducted using a JEOL JSM-6610 LV SEM (JEOL USA Inc., Peabody, MA, USA) scanning electron microscope at the Shared Instrumentation Facility at Louisiana State University. Small fabric swatches were mounted on stubs, coated with gold/palladium, and image scanned under high vacuum with an accelerated voltage of 5 kV using an Everhart–Thornley detector (ETD; JEOL, USA Inc.).

### 2.6. Absorption Capacity

The absorption capacity was measured on a GESTER GT-CNO2 Nonwovens Absorption Tester XINJE (Gester International Co., Ltd., Quanzhou, China). Fabric samples, 10 cm × 10 cm, were attached to a metal frame and lowered into a reservoir of distilled water at a rate set at 30 cm/min, leaving the sample and frame submerged for 60 s and then lifting the sample and frame from the reservoir at the same rate. When the frame and sample have reached their highest point above the reservoir, the frame/sample is allowed to drain for 120 s. The apparatus constantly records the combined weight of the frame, sample, and absorbed water during this interval. At the end of draining, the absorbency capacity is calculated; A% = ((W_f_ – W_i_)/W_i_) × 100%; W_f_ = final combined weight; and W_i_ = initial weight of fabric. 

## 3. Results

**Figure 1:**
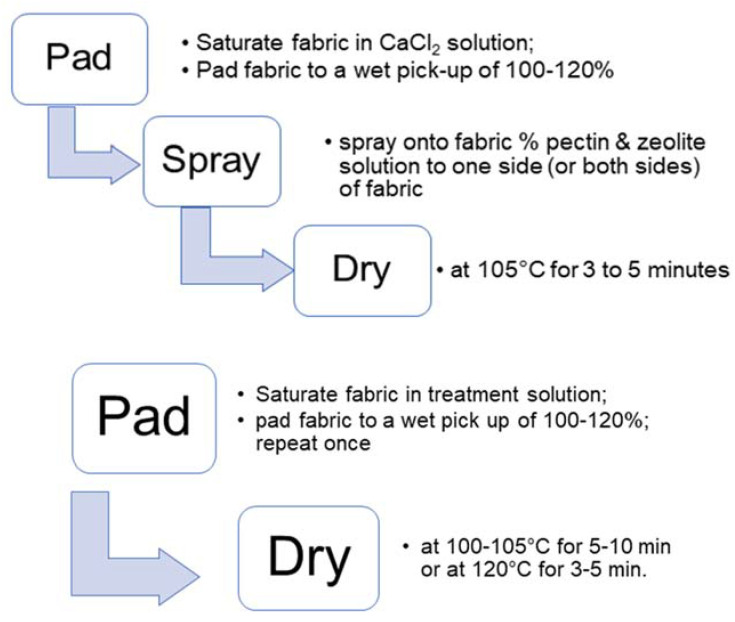
Diagram of finishing chemistry approaches to fabric treatment. The schematic emphasizes and details the differences applying the formulations to the fabric through a spray technology versus a pad and dry. In the case of Pad–Spray–Dry optimal wet pickup of the calcium chloride was realized by padding the fabric prior to the spraying step.

An examination of the effect of the zeolite-containing formulations on hemostasis was evaluated using TEG. The zeolite/pectin/calcium formulations were applied to TACGauze (TGz) and contrasted with analogous formulations on fine mesh gauze to assess the relative contribution of a nonwoven greige cotton fiber-containing substrate with a woven bleached cotton gauze. Two different approaches to the fabric finishing chemistry are contrasted with both dressing substrates as shown in Figure 1. The two-step application employs a pre-treatment with calcium chloride and pad-dry-cure application methods.

**Figure 2:**
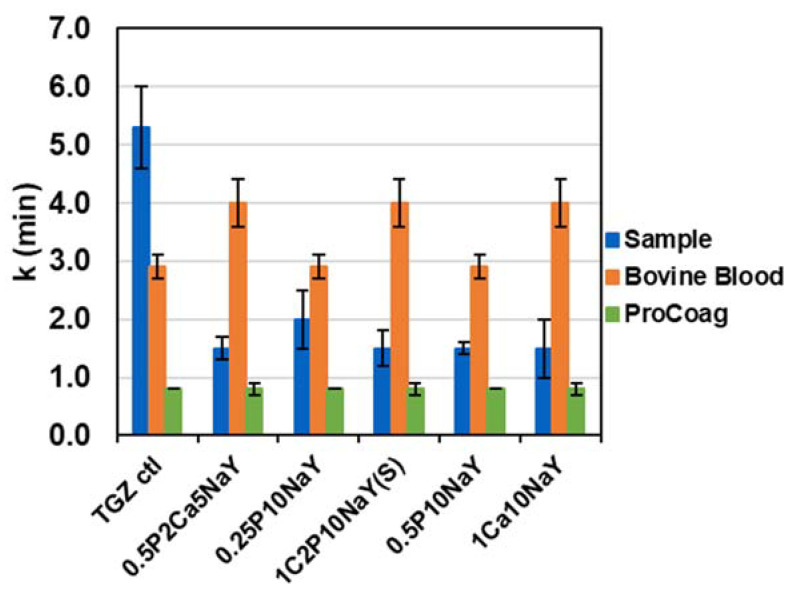
The time to clot (k) formation TEG data of separate TACGauze (TGz) swatches treated with the following formulations and controls presented in this graph consist of: (l.-r.) TACGauze untreated (TGz Ctl); 0.5% Pectin + 2% CaCl_2_ + 5% NaY (0.5P2Ca5NaY); 0.25% Pectin + 10% NaY (0.25P10NaY); 1% CaCl_2_: 2% Pectin + 10% NaY sprayed (1C2P10NaY(S)); 0.5% Pectin + 10% NaY (0.5P10NaY); and 1% CaCl_2_ + 10% NaY (1Ca10NaY). Error bars represent the standard deviation of duplicate determinations.

**Figure 3:**
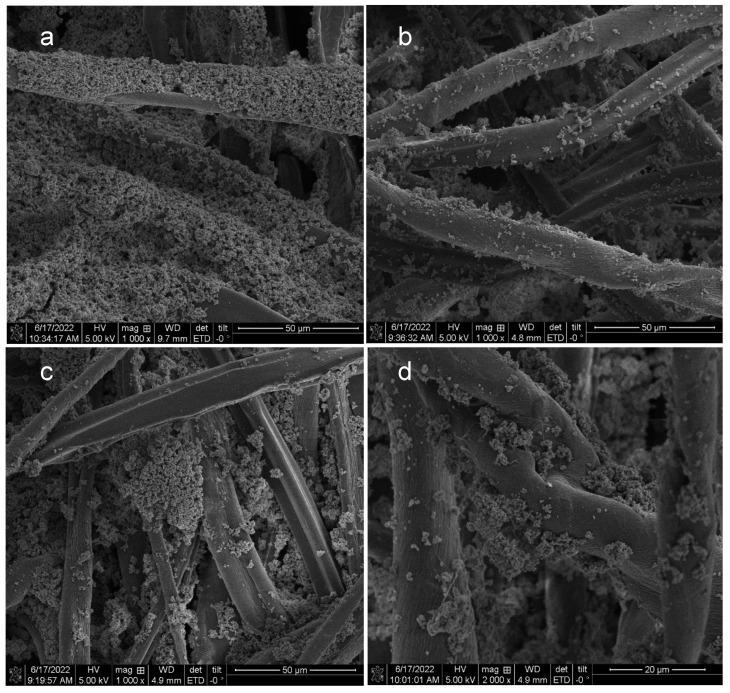
Scanning Electron Microscopy (SEM) micrographs taken of TGz treated fabric swatches with (left-right, top to bottom) (**a**) 1% CaCl_2_ (pad) + 2% pectin + 10% NaY zeolite (spray); (**b**) 1% CaCl_2_ + 10% NaY zeolite; (**c**) 0.5% pectin + 10% NaY zeolite(pad); and (**d**) 0.5% pectin + 10% NH_4_Y zeolite. Micrographs (**a**–**c**) are shown at 1000× magnification and (**d**) at 2000× magnification.

In Figure 2, it is apparent, based on comparing clotting time values, that the application of the formulation with a spray method resulted in comparable hemostatic activity to that of the pad dry method. Moreover, as shown in the SEM in Figure 3, the spray method yields a more even distribution across the network of entangled fibers of the fabric.

An assessment of the effect of calcium and pectin combined with the zeolite on accelerated clotting is also shown in Figure 2. A comparison between the time to clot formation in calcium/pectin formulations and pectin alone revealed a significant difference (based on ANOVA analysis summary) at *p* values that support statistically significant means (*p* < 0.05) to accelerated clotting when calcium is present. As noted below in the FTIR analysis of a formulation swatch, dried without fabric, a shift of the IR peak suggests exchange of calcium for sodium. This is consistent with the role that calcium plays as a co-factor in the coagulation cascade as is found in the conversion of prothrombin to thrombin and the formation of fibrin [18]. Previous observations have documented calcium release in blood as promoting blood coagulation [19]. Moreover, previous reports of dressings based on calcium modified starch have demonstrated activation of the intrinsic pathway of the coagulation cascade [20].

The effect of two zeolite counterion analogs on hemostatic activity was compared with sodium and ammonium forms of zeolite. The results of the effect of the counterion substitutions on fibrin and clot formation are shown in Table 1 and Table 2. Notably ammonium as a counterion resulted in shorter times to fibrin and clot formation comparable to the procoagulant standard. Moreover, the time to fibrin and clot formation for the ammonium analog was found to be within a range consistent with a procoagulant dressing that modulates severe hemorrhage control [21].

On the other hand, sodium as a counterion resulted in slightly longer times to fibrin formation (50–60 percent higher R values) when compared with a standard procoagulant, but time to clot formation was comparable to the procoagulant standard. Thus, it is apparent that the addition of calcium and pectin in combination with ammonium zeolite yields lower R and K values than analogous formulations with sodium zeolite.

The results in Table 2 also indicate a correlation between fabric add-on of the zeolite/pectin/calcium formulary and accelerated clotting as measured by both time to fibrin and clot formation. The SEM micrographs contrasting the TGz and fine mesh gauze formulations in Figure 3 are consistent with the higher add-ons. For example, a higher specific surface area is provided on the nonwoven fibers for deposition of the zeolite/pectin/calcium formulation when compared with the woven substrate.

A comparison of the absorption capacity of the dressing substrates shown in Table 3 revealed a four-fold higher level for TGz versus standard cotton gauze and is relevant to the ability of dressings to promote clotting [22].

The effect of forming calcium oxide in the hemostatic formulation to act synergistically with Zeolite Y was also examined by employing formulations that promote formation or directly incorporate calcium oxide [23]. The TEG results are shown in Table 4. A minimal but significant increase in clot strength (MA) was observed over the procoagulant dressing standard, but no added advantage was observed as judged in this study by the comparative R and K values.

### FTIR Analysis to Characterize Formulary Structure and Quantity versus Function

**Figure 4:**
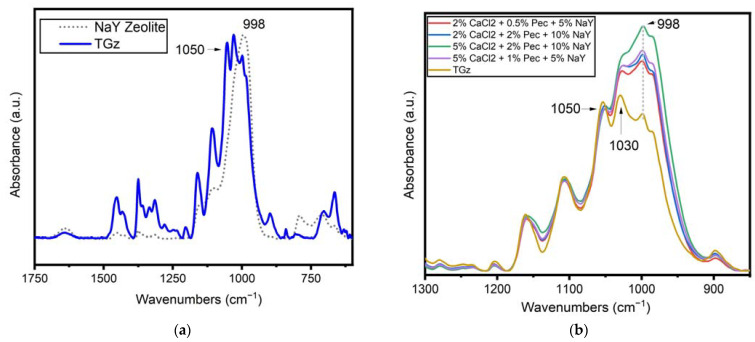
(**a**) FTIR spectra of TACGauze (TGz) (solid blue line) and NaY zeolite powder (gray dotted line). Spectra are presented normalized to their highest peak and as the average of four examination spots. (**b**) FTIR spectra of TGz fabrics treated with NaY zeolite, pectin, and CaCl_2_. An untreated TGz (control) is also shown. Spectra are presented normalized to 1050 cm^−1^ and as the average of four examination spots. A relative increase in the intensity of the 998 cm^−1^ peak is observed for the gauze fabrics treated with increasing NaY zeolite.

FTIR spectra shown in Figure 4a compare NaY zeolite powder and the principal untreated fabric, TGz. Notably, the NaY zeolite powder (gray dotted line) spectrum contains overlap in peak intensity absorbance with the TGz fabric (blue solid line). The highest peak for the zeolite is a wide band centered at 998 cm^−1^. Glycosidic orientations in the cellulose and other polysaccharides present in the TGz gauze contain four overlapping peaks in the same range. The noticeable difference in peak intensity of the 998 cm^−1^ peak relative to the 1050 cm^−1^ peak was used to monitor NaY zeolite uptake by treated TGz swatches.

The FTIR spectra of treated TGz gauze fabrics with formulations containing NaY, CaCl_2_ and pectin are shown in Figure 4b and are normalized to the peak near 1050 cm^−1^. This normalization allows for a simple comparison of the intensity of the zeolite 998 cm^−1^ peak previously discussed. As expected, the band near 998 cm^−1^ is observed as the most intense band in samples treated with the NaY zeolite. Moreover, the relative intensities of the 998 cm^−1^ band for the treated samples are consistent with formulary add-ons in Table 2. For example, the sample treated with 5% CaCl_2_, 2% pectin, and 10% NaY (green trace) gave an FTIR response consistent with the highest uptake of the NaY zeolite and overall percent add-on of 85.9%. The second highest relative intensity for the 998 cm^−1^ peak is observed for the other sample that used 5% of CaCl_2_ (purple trace), a sample that registered a 52.2% add-on. Interestingly, the samples that used 2% of the CaCl_2_ had slightly lower relative intensity to the 998 cm^−1^ peak, and slightly smaller add-ons percentages (between 40.4 and 48.7% add-on).

FTIR spectroscopy is also useful in confirming uptake of the ammonium zeolite in the dressing samples (Figures S1 and S2). Similar to the NaY formulations, an increase in the relative intensity of the 998 cm^−1^ band is observed for a gauze sample treated with NH_4_Y. Comparison of the relative intensity of the 998 cm^−1^ is more complicated for the NH_4_Y, in part due to the omission of CaCl_2_ or pectin from some of the samples examined. Nevertheless, the close relative intensity of the 998 cm^−1^ bands for the NH_4_Y formulations shown in Figure S1 are in line with the narrow percent add-on observed for the samples, from 35.3 to 46.7% add-on listed in Table 1.

**Figure 5:**
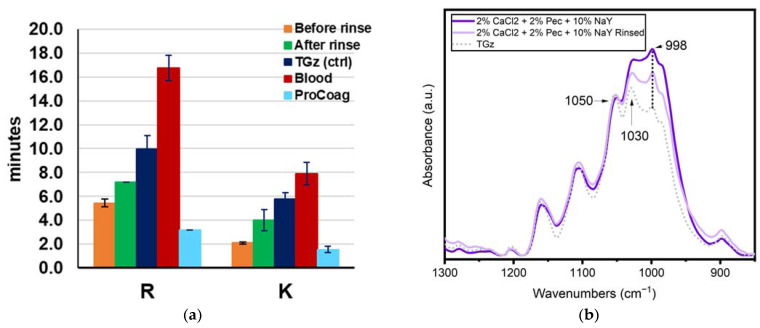
(**a**) Graphed time to fibrin (R) and clot (K) formation TEG data of TACGauze (TGz) treated, 0.5%Pectin + 2%CaCl_2_ + 5%NaY, before (orange) and after rinsing (green) with add-ons of 40.4% and 20.6%, respectively. Error bars represent the standard deviation of duplicate determinations. (**b**) FTIR spectra of a TGz fabric treated with NaY zeolite, pectin, and CaCl_2_, before and after rinsing. An untreated TGz (control) is also shown. Spectra are presented normalized to 1050 cm^−1^ and as the average of four examination spots.

To assess the adherence of the formulation components to the fabric, a comparison of fabric-formularies before and after rinsing was performed to remove what may be loosely adherent of the formulary. The hemostatic profiles of the formulations before and after rinsing are shown in Figure 5a. Rinsing of the TACGauze (TGz) treated with 0.5% Pectin + 2% CaCl_2_ + 5% NaY reduced the percent fabric add-on by 49%, which represents remaining formulation components adhering to the fabric. The subsequent TEG-determined R (time to fibrin formation) and K (time to clot formation) values before rinsing the fabric/formulations revealed a decreased time to fibrin formation by 45% and time to clotting by 63% compared with untreated blood. However, the rinsed fabric decreased time to fibrin formation by 28% and time to clot formation by 31%. Notably these differences in time to fibrin and clot formation are supported by ANOVA determined significantly different standard deviation at *p* < 0.05. FTIR was employed to monitor formulation retention to the fabric shown in Figure 5b before and after being rinsed. Spectral intensities are again presented normalized to the 1050 cm^−1^ peak. Notably the rinsed sample was free of non-adherent formulary powder from the treatment. Rinsing did reduce the relative intensity of the 998 cm^−1^ peak, yet the relative intensity was still higher than what was observed for the untreated gauze fabric. The decrease in the relative intensity of the 998 peak corresponds to about a 29% overall reduction. As shown in Figure 5b, the FTIR peak for the rinsed fabric is halfway between the untreated TGz fabric and the before rinse treated fabric. Thus, a portion of the NaY zeolite is retained in the fabric following rinsing. This is consistent with the approximate 50% loss in hemostatic activity noted above and with the TEG results discussed above and shown in Figure 5a. 

**Figure 6:**
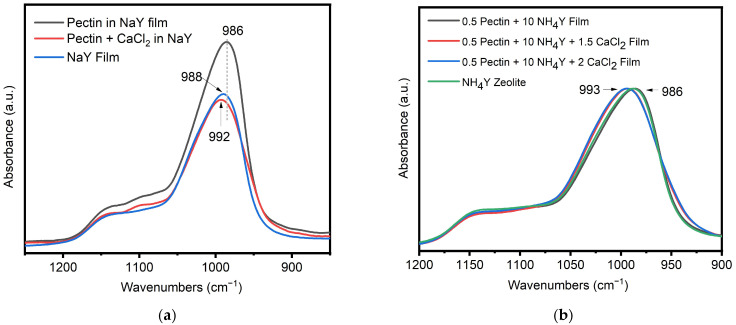
Formulary films of (**a**) sodium Y(NaY) or (**b**) ammonium Y (NH_4_Y)zeolite containing pectin and/or calcium compared to its respective powder.

An additional FTIR study was undertaken to explore interaction of the CaCl_2_ salt with the zeolites used in the present study. Films containing the NaY zeolite pectin and CaCl_2_ were used to model their interaction and are shown in Figure 6. For this study, the zeolite powder and films were dried overnight in an oven at 105 °C. This step was taken to reduce the impact of moisture uptake by the zeolites to impact the peaks positions. As such, the peak positions of the zeolite reported below differ slightly than those discussed in the previous sections. Notably, the position of the most intense band in the NaY zeolite powder changes when the zeolite made into a film containing pectin, a shift from 988 cm^−1^ for the zeolite on its own to 986 cm^−1^. Notably, the addition of CaCl_2_ further shifts the location of the peak to 993 cm^−1^. On average, a shift of peak position for the samples that contained CaCl_2_ to 993 cm^−1^ from 988 cm^−1^ is an indication that the CaCl_2_ is interacting with the zeolite. This shift is not only observed in the formulations alone. A small shift is also observed in the gauze treated with NaY formulations described above (Figure 4), with the peak for the zeolite powder on its own observed at 996 cm^−1^, while the gauze treated with the zeolite and CaCl_2_ showing the peak at 998 cm^−1^.

## 4. Discussion

The structure function relations performed on the fabrics and formulations of this study are based on their thromboelastographic (TEG) profiles and are consistent with our previously outlined approach on the evaluation of hemostatic cotton-based dressings where we employed TEG in the analysis of hemostatic materials [7,25,26]. Thromboelastography principally measures the intrinsic pathway (protein-based) of blood clotting which results in the conversion of fibrinogen to fibrin [13]. This is done by assessing the bloods viscoelastic properties under low shear conditions. Thus, the results of the thromboelastographic profiles are reported here as time to initial fibrin formation (R) and time to formation of a 20 mm clot (K).

The design of the fabric formulations was based on incorporation of zeolite Y as the principal procoagulant, with a low percentages of a calcium and pectin mix to adhere and enhance the activity in synergism with the dressing fiber composition [27]. Zeolite Y is a crystalline aluminosilicate with a unit cell containing AlO_4_^5−^ and SiO_4_^4−^ at the corners of a tetrahedron. The molecular structure is three dimensional and cage-like with an average pore size of 12.7 angstroms. Each aluminum oxide tetrahedron introduces a negative charge in the lattice. Thus counter-cations can occupy the pores by way of interacting with negatively charged oxo-aluminum centers [27].

### Comparison of the Effect of Zeolite Counterion Substitutions

In this study, zeolite Y was employed due to its high Si:Al ratio of five and Si:Ca associated with enhancing hemostasis [14]. The negatively charged surface of zeolites is also thought to play a role in contact activation by way of interaction with fibrinogen and Factor XII [4]. Since the binding of the counterions is rather weak, it is likely that calcium displaces the ammonium and sodium salts of the zeolites of this study, and ion exchange of sodium for calcium has been documented [28]. Moreover, this type of ion exchange also occurs with pectin to form the classic egg-box conformation characteristic of calcium bound pectin [29].

In this study, calcium and pectin were combined with two counterion forms of zeolite Y to promote adherence and enable hemostasis. The study also evaluates two different fabric finishing approaches on two fabric substrates and the overall effect of formulations, fabric, and finishing approaches on add-ons. Several notable results are discerned. For example, ammonium as a counterion improved times to fibrin and clot formation relative to the control. This is interesting considering the lower add-ons of formulary observed for the ammonium zeolite versus the sodium zeolite applications. However, an examination of the SEMs shown in Figure 3c illustrates a close packed aggregate structure of sodium zeolite within the inter-fiber spaces suggesting that a high degree of zeolite aggregation gives a lower surface area for contact activation hemostasis to occur when compared with the ammonium zeolite. This is more evident when compared with the SEMs of the ammonium zeolite formulation in Figure 3d.

Another feature concerning the fabric structure/function relationship is the role that the absorption capacity of TGz plays in hemostasis. The enhanced absorption capacity of TGz has been shown to influence hemostatic activity byway of the design of a balance of hydrophobic and hydrophilic fiber blends [25]. Absorption capacity does not directly correlate with hemostasis in the case of all dressing types, but in nonwovens it has been shown to play a decided role of influencing rate to hemostasis [19,22,25,30]. Here it is thought to influence the hemostatic activity of the zeolite formulary by retention and diffusion of blood platelets and intrinsic coagulation factors within the fibrous network of the nonwoven fabric.

It was also found that FTIR analysis using peak differential fitting facilitated a correlation between peak intensity and fabric add-ons. Thus, this approach should be useful in assessing fabric uptake of zeolite onto fabric surfaces. This was evident with both ammonium and sodium zeolite.

Identification of zeolite counterion exchange was made based on an FTIR signature peak frequency shift associated with calcium present in the formulation for sodium zeolite to form calcium zeolite. This is seen in the symmetrical stretching frequency for counterion exchange of forms of zeolite Y [28]. For example, in this study the observed shift from 989 cm^−1^ for sodium zeolite to 992 cm^−1^ for calcium. The shift from a lower to higher stretching frequency is consistent with calcium forming a stronger interaction with the zeolite pore by way of the two electrons in the 2 s orbital versus sodium with a 1 s electron. In addition, an observed shift to a lower vibrating stretching frequency (989 to 986 cm^−1^) occurs with the addition of pectin. Thus, an interaction of pectin with sodium zeolite is apparent. This suggests that sodium zeolite interacts with the pectic acid present in the formulation. This is consistent with the close-packed zeolite crystal aggregates observed in the SEM shown in Figure 3c.

As previously reviewed carbohydrates have been reported for enhancing hemostasis [31], zeolite-loaded, alginate-chitosan, pectin-cellulose hydrogels, and zeolite-starch complexes have been examined for hemostatic activities [20]. Pectin possesses muco-adhesive properties [26] and was applied in the formulary of this study for that purpose. Pectin promotes formation of hydrogels and has been employed to create dressing structure motifs for chronic and acute wounds [32,33]. Calcium also interacts with pectin as noted above and with pectin resides in the primary cell wall of cotton. Thus, one design objective of the study was to adhere pectin to the exposed primary cell wall of the cotton fiber with the cotton fiber pectin residing there. The SEM micrographs also indicated deposition of a calcium/pectin residue formed on the surface of the zeolite crystals. We have previously suggested the importance of the design motif in greige cotton-based dressings [26]. 

Calcium carbonate and calcium oxide have been shown to promote hemostasis. The exogenous availability of calcium in the form of salts and metal oxides within the acute wound has been associated with improved clotting outcomes as judged by rate and strength of clotting [23]. This is attributed to providing an enhancement of the intrinsic pathway of the coagulation cascade since calcium serves as a co-factor to facilitate enzymatic formation of fibrin [18]. We observed a slight increase in clot strength but not to the extent observed by Ostomel. Likewise, several reports have demonstrated how calcium when attached to microporous starch and zeolite facilitated enhanced clotting [20,34]. Thus, the formulary treatment with calcium in this study results in exchange of counterions to some extent and may further potentiate the release of calcium into blood upon exchange with sodium and potassium as observed by others [19].

## 5. Conclusions

This study portrays the structure/function relations of zeolite/pectin/calcium chloride formulations applied to a highly absorbent hemostatic nonwoven blend of cotton fibers. The importance of employing counterion exchange to promote improved hemostatic activity is underscored in this study, and the application of pectin and calcium chloride to promote activity and adhesion is shown to be compatible with the greige cotton fiber composition. The use of FTIR to characterize zeolite formularies has been shown in this study to be applicable to characterizing both quantitative uptake and counterion exchange. The study demonstrates the further potential of employing key fabric substrates to promote hemostasis of procoagulant formularies.

## Figures and Tables

**Table 1:** Percent add-on and TEG data ^a^ of pad-dry application of pectin, CaCl_2_, and NH_4_Y zeolite on TGz.

Sample Description ^b^	%Add -On	R (min)	σ	K (min)	σ
10% (*w*/*v*) NH_4_Y only	33.8	5.1	0.8	2.2	1.0
1.5% CaCl_2_ + 10% NH_4_Y	35.3	5.7	0.3	3.2	1.5
0.25% PECTIN + 10% NH_4_Y	36.2	5.4	0.3	2.8	0.8
0.5% PECTIN + 10% NH_4_Y	37.5	5.2	0.4	2.4	0.5
1.0% PECTIN + 10% NH_4_Y	41.6	6.5	0.4	2.6	1.0
0.5% PEC+ 1.5% CaCl_2_ + 10% NH_4_Y	46.7	5.9	0.5	2.0	0.3
TGz (untreated)		14.4		6.5	
Blood (bovine)		19.1	1.8	10.7	2.3
Procoagulant		5	0.3	2.4	0.7

^a^ The time to fibrin(R) and clot (K) formation. ^b^ Abbreviations and definitions of variables included in the table are PEC = pectin, NH_4_Y = Ammonium Y zeolite, CaCl_2_ = calcium chloride, TGz = TACGauze, and the standard deviation, σ. Standard deviation was calculated for duplicate determinations.

**Table 2:** Percent add-on and TEG data ^a^ of pad-dry application of pectin, Calcium chloride (CaCl_2_) and zeolite on TGz and fine mesh gauze.

Sample Description ^b^	%Add -On	R (min)	σ	K (min)	σ
TGz 0.5% PEC + 2% CaCl_2_ + 5% NaY (1)	40.4	5.2	0.4	1.8	0.2
TGz 2% CaCl_2_, 2% PEC +10% NaY (2)	48.7	6.4	1.0	2.7	1.1
TGz 5% CaCl_2_, 2% PEC +10% NaY (2)	85.9	5.6	0.3	2.2	0.6
TGz 5% CaCl_2_, 1% PEC + 5% NaY (2)	52.2	5.8	0.1	2.0	0.3
FMGz 0.5% PEC + 2% CaCl_2_ + 5% NaY (1)	22.4	6.8	0.5	3.9	0.3
FMGz 2% CaCl_2_, 2% PEC +10% NaY (2)	29.4	6.8	0.1	4.6	1.1
FMGz 5% CaCl_2_, 2% PEC + 10% NaY (2)	37.2	7.2	0.0	3.0	0.4
FMGz 5% CaCl_2_, 1% PEC + 5% NaY(2)	21.2	7.4	0.6	4.6	1.1
TGz (untreated)	-	8.1	0.6	3.8	0.1
Blood (bovine)	-	14.9	1.4	6.2	1.3
Procoagulant	-	3.4	0.2	1.1	0.1

^a^ The time to fibrin(R) and clot (K) formation. ^b^ Abbreviations and definitions of variables included in the table are: (1) = one-step pad-dry; (2) = two-steps: CaCl_2_ padded and dried then padded pectin + zeolite solution and dried; (S) = sprayed application of zeolite; TGz = TACGauze, FMGz = fine mesh gauze, bleached 100% cotton, PEC = pectin, NaY = sodium Y zeolite, and the standard deviation, σ. Standard deviation was calculated for duplicate determinations.

**Table 3:** Absorption capacity of fabric dressing base.

Fabric	Absorption ^a^ Capacity %	STD	%CV
TACGauze (TGz)	852.17	41.6	4.88
100% Cotton Fine Mesh Gauze	286.86	25.4	8.86

^a^ The absorption capacity% is the average of five determinations, STD = standard deviation.

**Table 4:** TEG Data ^a^ and %add-on of calcium oxide (CaO) nanoparticle coated cotton with NaY zeolite on TGz.

Sample Description ^b^	%Add -On	R (min)	σ	K (min)	σ	MA (deg)	σ
CaO/SDS + 1% NaY ^c^	4.9	7.2	0.9	4.1	0.4	52.1	1.4
CaO/SDS + 5% NaY ^c^	19.5	6.2	0.3	3.2	0.3	53.9	1.8
Na_2_CO_3_, CaCl_2_, 10% NaY ^d^	31.9	6.4	0.5	4.0	0.6	51.4	2.7
NaOH, CaCl_2_, 10% NaY ^d^	30.9	6.2	0.4	3.3	0.0	53.3	2.5
Blood (bovine)		13.7	0.9	5.9	0.3	53.2	1.7
Procoagulant		4.0	0.4	3.1	0.4	49.6	2.4

^a^ The time to fibrin(R) and clot (K) formation and MA = clot strength. ^b^ Abbreviations and definitions of variables included in the table are Calcium chloride (CaCl_2_), sodium carbonate (Na_2_CO_3_), calcium oxide (CaO), sodium hydroxide (NaOH), sodium dodecyl sulfate (SDS), and the standard deviation, σ. Standard deviation was calculated for duplicate determinations. ^c^ Samples were treated with 0.2NaOH, 0.1CaCl_2_, and 1 mM of SDS to form the CaO nanoparticles following modified procedure (unpublished results). ^d^ These samples were made from modified procedure based on other published work [24].

## Data Availability

Not applicable.

## Supplementary Materials

The following supporting information can be downloaded at: https://www.mdpi.com/article/10.3390/jfb14050255/s1, Figure S1 FTIR spectra of TGz fabrics treated with NH_4_Y zeolite and various amounts of the pectin and CaCl_2_; Figure S2 FTIR spectra of a TAGz gauze (black trace) and of NH_4_Y zeolite on the gauze (red trace); and Figure S3 FTIR of NH_4_Y zeolite (10%) and pectin (0.5%) films with and without addition of calcium chloride, 1.5 and 2% respectively, and the zeolite powder control.
