# Supplementary material for: A Comparison of Hemostatic Activities of Zeolite-Based Formulary Finishes on Cotton Dressings
Source: J Funct Biomater. 2023 May 4;14(5):255. doi: 10.3390/jfb14050255 (PMC10219473; doi:10.3390/jfb14050255)
Supplement: Supplementary file 1 [file jfb-14-00255-s001.zip › jfb-2226151-SI.pdf]

Supplementary

A Comparison of Hemostatic Activities of Zeolite-Based Formulary Finishes on Cotton Dressings

J. Vincent Edwards *, Nicolette T. Prevost and Michael Santiago Cintron

Southern Regional Research Center, United States Department of Agriculture (USDA), Agricultural Research Service, New Orleans, LA 70124, USA

* Correspondence: vince.edwards@usda.gov

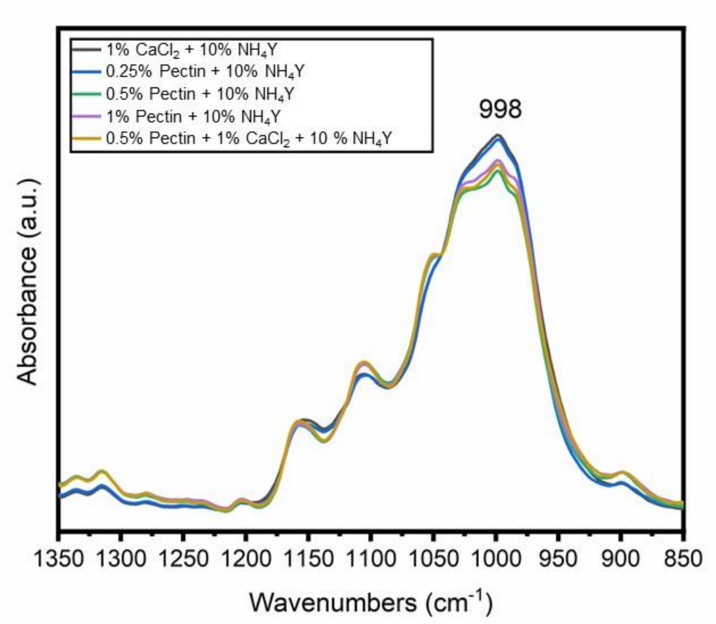

Figure S1. FTIR spectra of TGz fabrics treated with NH_4Y zeolite and various amounts of the pectin and $CaCl_2$. Spectra are presented normalized to 1050 cm^{-1} and as the average of 4 examination spots. A relative increase in the intensity of the 998 cm^{-1} peak is observed for gauze fabrics following treatment with NH_4Y zeolite and one other component.

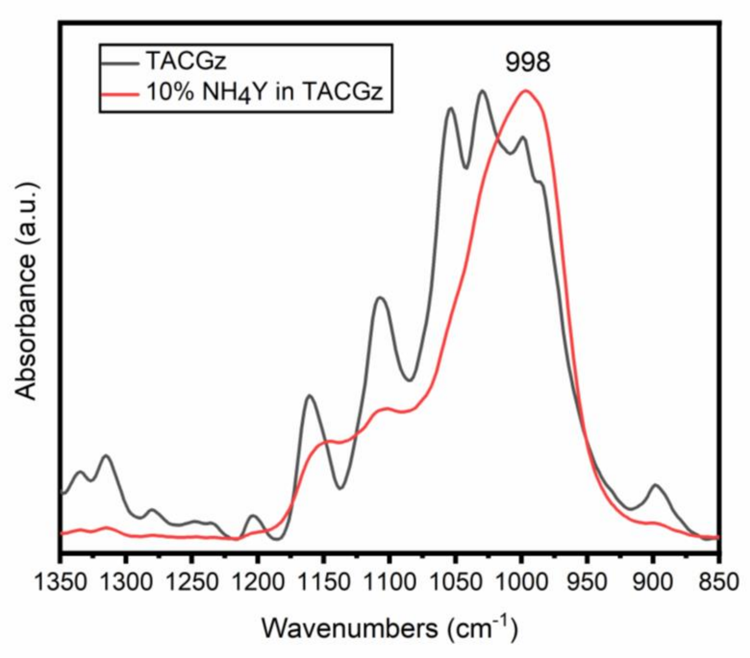

Figure S2. FTIR spectra of a TACGz gauze (black trace) and of NH_4Y zeolite on the gauze (red trace). Spectra are presented normalized to their highest peak, and as the average of 4 examination spots.

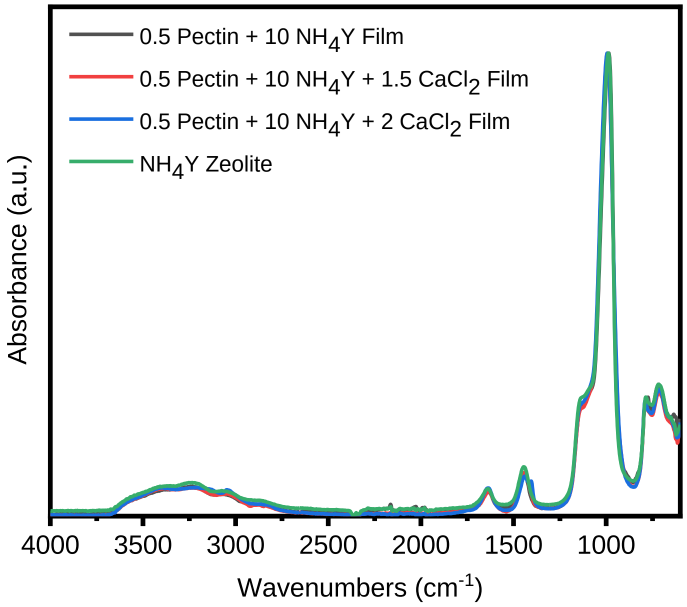

Figure S3. FTIR of ammonium Y zeolite(10%) and pectin(0.5%) films with/without addition of calcium chloride 1.5% and 2% respectively, and the zeolite powder control. This is the full spectrum of 6b.
